# Intravenous versus oral etoposide: efficacy and correlation to clinical outcome in patients with high-grade metastatic gastroenteropancreatic neuroendocrine neoplasms (WHO G3)

**DOI:** 10.1007/s12032-018-1103-x

**Published:** 2018-03-06

**Authors:** Abir Salwa Ali, Malin Grönberg, Seppo W. Langer, Morten Ladekarl, Geir Olav Hjortland, Lene Weber Vestermark, Pia Österlund, Staffan Welin, Henning Grønbæk, Ulrich Knigge, Halfdan Sorbye, Eva Tiensuu Janson

**Affiliations:** 1 Department of Medical Sciences, Section of Endocrine Oncology, Uppsala University, Uppsala, Sweden; 2 Departments of Surgery C and Endocrinology PE, Rigshospitalet, Faculty of Health Science, University of Copenhagen, Copenhagen, Denmark; 3 Department of Oncology, Copenhagen University Hospital Rigshospitalet, Copenhagen, Denmark; 4 Department of Oncology, Aarhus University Hospital, Aarhus, Denmark; 5 Department of Oncology, Oslo University Hospital, Oslo, Norway; 6 Department of Oncology, Odense University Hospital, Odense, Denmark; 7 Department of Oncology, Tampere University Hospital and Tampere University, Tampere, Finland; 8 Department of Oncology, Helsinki University Hospital and Helsinki University, Helsinki, Finland; 9 Departments of Hepatology and Gastroenterology, Aarhus University Hospital, Aarhus, Denmark; 10 Department of Oncology, Haukeland University Hospital, Bergen, Norway

**Keywords:** Chemotherapy, Intravenous, Oral, Etoposide, Neuroendocrine neoplasms, WHO G3

## Abstract

High-grade gastroenteropancreatic neuroendocrine neoplasms (GEP-NENs, G3) are aggressive cancers of the digestive system with poor prognosis and survival. Platinum-based chemotherapy (cisplatin/carboplatin + etoposide) is considered the first-line palliative treatment. Etoposide is frequently administered intravenously; however, oral etoposide may be used as an alternative. Concerns for oral etoposide include decreased bioavailability, inter- and intra-patient variability and patient compliance. We aimed to evaluate possible differences in progression-free survival (PFS) and overall survival (OS) in patients treated with oral etoposide compared to etoposide given as infusion. Patients (*n* = 236) from the Nordic NEC study were divided into three groups receiving etoposide as a long infusion (24 h, *n* = 170), short infusion (≤ 5 h, *n* = 33) or oral etoposide (*n* = 33) according to hospital tradition. PFS and OS were analyzed with Kaplan–Meier (log-rank), cox proportional hazard ratios and confidence intervals. No statistical differences were observed in PFS or OS when comparing patients receiving long infusion (median PFS 3.8 months, median OS 14.5 months), short infusion (PFS 5.6 months, OS 11.0 months) or oral etoposide (PFS 5.4 months, OS 11.3 months). We observed equal efficacy for the three administration routes suggesting oral etoposide may be safe and efficient in treating high-grade GEP-NEN, G3 patients scheduled for cisplatin/carboplatin + etoposide therapy.

## Introduction

Gastroenteropancreatic neuroendocrine neoplasms (GEP-NENs) with proliferation index (Ki67) > 20% and/or mitoses > 20 per 2 mm^2^ are aggressive tumors that belong to the Grade 3 (G3) group in the WHO classification of tumors of the digestive system with an expected 5-year survival of 16% [1–3]. The majority of these tumors are characterized as poorly differentiated and called neuroendocrine carcinomas (NECs) although a subgroup of well-differentiated G3 tumors has been recognized recently [4]. In the most recent WHO classification, pancreatic well-differentiated NENs G3 are denoted pancreatic NET-G3 [5].

GEP-NENs G3, excluding pulmonary NENs, account for approximately 50% of all NENs G3 with primary tumors in esophagus, stomach, pancreas, colon and rectum. However, they present with cancer of unknown primary (CUP) in 30% of the cases [6]. An increase in incidence has been observed during the last years. However, treatment efficacy does not seem to improve at the same rate [3, 7]. In the Nordic NEC study of 305 patients, median overall survival (OS) was 11 months for patients treated with chemotherapy and 1 month for untreated patients. Pancreatic tumors showed a median OS of 15 months, while rectal and colon tumors had median OS of 10.0 and 8 months, respectively, indicating that OS differs with primary tumor location [6, 8]. Other reported factors indicating a better prognosis are Ki67 index < 55%, normal serum lactate dehydrogenase (LDH) and platelet count as well as good performance status [6].

Platinum-based combination chemotherapy with cisplatin/carboplatin and etoposide is the first-line treatment for GEP-NENs [9, 10]. This treatment regimen has been agreed upon and is used by the Nordic, European and North American Societies of neuroendocrine tumors [11–13]. Clinical practices on route of administration of etoposide for these GEP-NEN patients differ and are mainly due to hospital specific recommendations.

Chemotherapy has for years been given through an intravenous (IV) route of administration for a variety of cancers although there has been a rise in oral anticancer drugs [14]. There is a general perception that anticancer drugs are best given intravenously but this has been challenged by the development of new formulations of drugs with increased stability and bioavailability. The growing number of alternatives in oral formulations is giving rise to a shift in attitudes and practices but still concerns remain [14, 15].

IV administration of etoposide holds several advantages. The ability of etoposide to exert its topoisomerase inhibitory effect is directly correlated to the concentration and duration of etoposide in the blood [16]. IV formulations allow for a higher bioavailability of a drug in the blood stream and therefore have been seen as the preferred route of administration but this also comes with disadvantages such as long hospital stays and elevated costs for hospitals as well as impact on patients’ quality of life. Another disadvantage with IV administration is the use of solvents that are toxic upon repeated administration [15–17].

Oral etoposide is an alternative still being debated. Concerns of using oral etoposide include the decreased bioavailability, inter- and intra-patient variability as well as for the risk of decreased patient compliance [14, 17]. Most studies show a bioavailability of etoposide ranging between 30 and 76% with a nonlinear absorption that decreases in bioavailability with increased dosage.

In this study, we examined the differences in treatment efficacy measured as progression-free survival (PFS) and OS for patients receiving etoposide as short or long infusions (≤ 5 or 24 h) compared to oral etoposide (O.E.).

## Materials and methods

### Patient and tumor characteristics

This study cohort included patients diagnosed with a GEP-NEN G3 with a primary tumor located in esophagus, stomach, pancreas, colon, rectum and a subgroup of CUPs (Table 1). CUP was defined as a NEN G3 diagnosed in a patient with predominant abdominal metastases but where no primary tumor could be identified.

**Table 1:** Patient and baseline disease characteristics

	IV 24 h, *n* (%)	IV ≤ 5 h, *n* (%)	O.E., *n* (%)
	170	33	33
*Sex*			
Male	86 (51)	20 (61)	18 (55)
Female	84 (49)	13 (39)	15 (45)
*Primary tumor*			
Esophagus	6 (3)	2 (6)	0 (0)
Stomach	10 (6)	2 (6)	4 (12)
Pancreas	48 (28)	8 (24)	10 (31)
Colon	35 (21)	7 (21)	4 (12)
Rectum	12 (7)	3 (9)	3 (9)
CUP	59 (35)	11 (34)	12 (36)
*Chromogranin A immunoreactivity*			
Positive	142 (86)	24 (80)	26 (85)
Negative	23 (14)	6 (20)	4 (15)
*Synaptophysin immunoreactivity*			
Positive	153 (94)	33 (100)	29 (94)
Negative	9 (6)	0 (0)	2 (6)
*Performance status*			
0	59 (37)	11 (34)	8 (25)
1	83 (52)	16 (48)	17 (53)
2	14 (9)	5 (15)	6 (19)
3	3 (2)	1 (3)	1 (3)
*LDH*			
Normal	55 (41)	11 (33)	18 (62)
Elevated	80 (59)	22 (67)	11 (38)
*Metastatic stage*			
Local	7 (4)	3 (9)	1 (3)
Regional	41 (24)	4 (12)	6 (18)
Distant	122 (72)	26 (79)	26 (79)
*Ki67*			
< 55%	79 (46)	19 (58)	17 (52)
≥ 55%	91 (54)	14 (42)	16 (48)
*Response*			
Complete	5 (3)	0 (0)	2 (7)
Partial	41 (26)	8 (29)	8 (27)
Stable disease	59 (37)	10 (35,5)	13 (43)
Progressed disease	54 (34)	10 (35.5)	7 (23)

Performance status: *ECOG* the Eastern Cooperative Oncology Group consensus for performance status, *Response* RECIST criteria, *IV* intravenous, *LDH* lactate dehydrogenase, *O.E.* oral etoposide

Patients were collected from the Nordic NEC study, resulting in 236 patients, diagnosed 1995–2012, in whom the route of etoposide administration could be verified. Clinical data were obtained from the Nordic NEC register [6]. The cohort included patients that received platinum-based combination chemotherapy (cisplatin/carboplatin + etoposide); cisplatin was given as an infusion to all 236 patients. Patients were given etoposide as short infusion (≤ 5 h), long infusion (24 h), or oral tablet (O.E.). The choice of route of etoposide administration was based on hospital preferences, and thus, all patients at a certain hospital were treated in the same way.

All tumors were immunoreactive (IR) for synaptophysin and/or chromogranin A (CgA) and all tumors had Ki67 index ≥ 20% counted in hot spot areas.

### Statistical analyses

Efficacy of etoposide in the three groups was assessed with regard to PFS and OS. The defined event was death from tumor progression. PFS was defined as the time from the first treatment until time of the first progression and OS was defined as the time from diagnosis of NEN G3 until date of death from neuroendocrine cancer; or if event was not found, censored at date of last observation.

Kaplan–Meier plots were used for PFS and OS analysis, and the log-rank test was used to compare curve separation according to type of etoposide administration. Cox proportional regression was performed for the estimation of hazard ratios (HRs) and confidence intervals (CIs). The two groups receiving IV infusions were compared to patients receiving O.E. Using a multivariate regression model, variables hypothesized to correlate with the clinical outcome were included to ensure that they would not confound with the analysis. These analyses were performed on dichotomized variables: sex (male vs. female), CgA (positive vs. negative), performance status ECOG (0 + 1 vs. 2 + 3), LDH (normal vs. elevated) and Ki67 (< 55 vs. ≥ 55%). All statistical analyses were performed using IBM SPSS statistics software (v25, USA).

### Ethics

The study was approved and the need for consent was waived by the local ethics committee, Regionala etikprövningsnämnden (EPN), in Uppsala, Sweden (ref: 2008/397).

## Results

### Baseline patient characteristics

Of the 236 patients included, 170 (72%) were given long infusions, oral etoposide and short infusions were given to 33 patients, respectively (Table 1). The median age in the whole cohort group was 61 years (range 27–85 years). Ki67 index was > 55% in 54% of all the patients. All included patients died due to their malignant disease. The distribution of variables such as sex, performance status, CgA immunohistochemical expression, LDH levels, metastatic disease and treatment response was similar in all three groups. Adverse events were not recorded in the Nordic NEC registry, and hence, analysis of the safety profile could not be performed. Patient and disease characteristics are presented in Table 1.

### Survival data

**Fig. 1:**
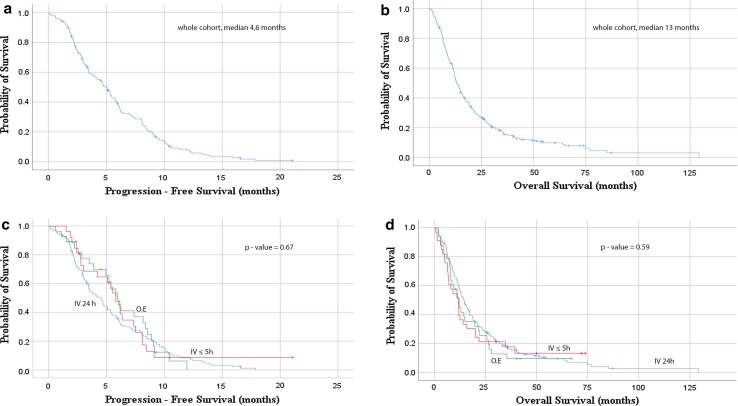
Kaplan–Meier curves for PFS and OS. **a** PFS for whole cohort; **b** OS for whole cohort; **c**, **d** PFS and OS for the three administrations, intravenous long infusion (IV 24 h), intravenous short infusion (IV < 5 h) and oral etoposide (O.E.)

PFS and OS showed no differences between the three groups. Median PFS for the whole cohort was 4.6 months and OS 13 months (Fig. 1a, b). Median PFS for the IV 24 h was 3.8 months, 5.6 months for 5 h infusion and 5.4 months for oral treatment. OS was also similar between the three groups; 14.5 months for IV 24 h, 11 months for 5-h infusion and 11.3 for oral administration.

### Association between administration and prognosis

Kaplan–Meier analysis for the three administrations did not show differences in PFS and OS (Fig. 1c, *p* = 0.67, Fig. 1d, *p* = 0.59). Additional analyses, comparing survival curves for 3 and 6 months, showed no differences in the short-term progression between the three groups (data not shown).

To assess the effect of administration on survival, cox regression models for univariate and multivariate analyses were used. We found no differences in progression and OS on the basis of administration when comparing oral administration to short and long infusion (Table 2, *p* = 0.54 and *p* = 0.91 for PFS and *p* = 0.38 and *p* = 0.90 for OS). A worse performance status and elevated LDH were significantly associated with poorer survival. Patients with performance status 2 + 3 showed an almost doubled risk of progression (*p* = 0.01) compared with patients with performance status 0 and 1 and almost four times poorer survival (*p* < 0.01). Elevated LDH levels also associated significantly to shorter time to progression and poorer survival in this patient cohort. Patients with elevated LDH level had a 50% higher risk of progression and 70% risk of dying (*p* = 0.01 and *p* < 0.01, respectively). Data are summarized in Table 2.

**Table 2:** Univariate analysis

	Progression-free survival		Overall survival	
	HR (95% CI)	*p* value	HR (95% CI)	*p* value
*Administration*				
IV ≤ 5 h versus O.E.	1.2 (0.7–1.8)	0.54	0.8 (0.6–1.2)	0.38
IV 24 h versus O.E.	1.0 (0.5–1.7)	0.91	1.0 (0.6–1.6)	0.90
*Sex*				
Male versus female	1.0 (0.7–1.3)	0.92	0.9 (0.7–1.2)	0.65
*Ki67*				
< 55 versus ≥ 55%	1.0 (0.7–1.3)	0.92	1.3 (0.9–1.7)	0.12
*CgA immunoreactivity*				
Positive versus negative	1.0 (0.7–1.5)	0.92	1.3 (0.9–1.9)	0.23
*ECOG performance status*				
0 + 1 versus 2 + 3	1.8 (1.2–2.8)	0.01**	3.8 (2.5–5.7)	<0.01**
*LDH*				
Normal versus elevated	1.5 (1.1–2.1)	0.01**	1.7 (1.3–2.4)	<0.01**
*Metastatic stage*				
Local versus distant	1.0 (0.4–2.6)	0.96	0.9 (0.5–1.8)	0.76
Regional versus distant	0.9 (0.4–2.4)	0.95	1.0 (0.5–2.0)	0.91

Hazard ratio (HR) and 95% confidence intervals (CI) obtained from cox regression models

*CgA* chromogranin A, *ECOG* the Eastern Cooperative Oncology Group consensus for performance status, *IV* intravenous, *LDH* lactate dehydrogenase, *O.E.* oral etoposide

*Correlation is significant at the 0.05 level

**Correlation is significant at the 0.01 level

In the multivariate model adjusted for sex, Ki67, CgA, performance status, LDH and metastatic stage, no statistical associations between administration route and progression/survival were found (Table 3).

**Table 3:** Multivariate analysis

	Progression-free survival		Overall survival	
	HR (95% CI)	*p* value	HR (95% CI)	*p* value
*Administration*				
IV ≤ 5 h versus O.E.	1.2 (0.7–2.2)	0.42	1.1 (0.6–1.8)	0.79
IV 24 h versus O.E.	1.0 (0.5–1.9)	0.99	1.1 (0.6–1.9)	0.86
*Sex*				
Male versus female	0.9 (0.7–1.3)	0.71	0.8 (0.6–1.1)	0.22
*Ki67*				
< 55 versus ≥ 55%	1.1 (0.8–1.6)	0.57	1.3 (0.9–1.8)	0.17
*CgA immunoreactivity*				
Positive versus negative	0.7 (0.4–1.1)	0.13	0.8 (0.5–1.3)	0.35
*ECOG performance status*				
0 + 1 versus 2 + 3	1.9 (1.2–3.3)	<0.01**	3.8 (2.4–6.2)	<0.01**
*LDH*				
Normal versus elevated	1.4 (0.7–2.0)	0.07	1.6 (1.1–2.2)	0.02*
*Metastatic stage*				
Local versus distant	1.1 (0.4–2.9)	0.88	1.2 (0.6–2.6)	0.62
Regional versus distant	0.9 (0.4–2.5)	0.91	1.2 (0.6–2.5)	0.59

Hazard ratio (HR) and 95% confidence intervals (CI) obtained from cox regression models

*CgA* chromogranin A, *ECOG* the Eastern Cooperative Oncology Group consensus for performance status, *IV* intravenous, *LDH* lactate dehydrogenase, *O.E.* oral etoposide

*Correlation is significant at the 0.05 level

**Correlation is significant at the 0.01 level

## Discussion

In this large Nordic NEC register-based study, we examined differences in disease progression and survival for patients receiving etoposide as 24 h infusions, shorter infusions or O.E. The main finding is that there was no statistically significant differences between the three administration groups, which suggest that no one of these administration routes is superior to another.

The cox regression analyses showed that none of the administration groups in particular were associated to better or poorer PFS or OS. Additional analyses to further exclude the risk of confounding factors showed no significant differences between the three groups.

The data from this study suggest that, although more frequently used, IV formulations may not be the ultimate route of administration.

Studies of head-to-head comparisons between IV etoposide and O.E. are limited with conflicting data. Some studies suggest that O.E. is better or at least equally effective as IV etoposide and some studies report the opposite. O.E. given to patients with ovarian cancer and prostate cancer as the second-line treatment showed the same or better efficacy and safety as IV administration [18, 19]. In a study on patients with castration-resistant prostate cancer, the efficacy, compliance and safety profile did not differ between etoposide given IV versus orally [19]. These results suggest, in accordance with our results, that O.E. may be a valuable option to consider when treating patients with etoposide.

A study on patients with non-small cell lung cancer demonstrated that the safety profile for O.E. is significantly better to that of IV etoposide. In this cohort, there was a significantly higher need for hospitalization due to neutropenia in the IV group compared to the oral group [20].

However, results favoring IV formulations have also been demonstrated. There are limitations of oral chemotherapy that may be the cause of their slow implementation in the clinic, such as variability in concentrations, uptake and safety profile. A study showed that O.E. has three times higher intra-patient variability and twice as high inter-patient variability when compared to IV formulation of the drug [17]. How important these inter- and intra-patient variabilities are for the survival of the patients is not concluded. Drug–drug interaction is another concern when giving etoposide orally. Etoposide interacts with the commonly used antifungal agent, ketoconazole, resulting in an increase in the etoposide concentration systemically [21].

The oral administration route for etoposide is an easier, by patients often preferred approach rather than IV infusions. An 89% preference for oral chemotherapy compared to IV was reported in a study from 1997 and in a meta-analysis of 13 research papers in 2016, the reported preference for oral chemotherapy was 85%. These results indicate a strong patient preference for oral administration, with no clear studies refuting the use of oral chemotherapy [22, 23]. Oral chemotherapy also decreases the cost associated with hospital stays for infusions, and it decreases the pains of puncture wounds in patients and offers a more convenient alternative. The cost effectiveness of oral chemotherapy has been investigated in few studies and many times with favorable outcome for the oral formulations [24, 25].

In conclusion, there are no conclusive results to indicate which route of administration of etoposide is superior. Our results indicate that the three ways of administrating etoposide did not differ with regard to efficacy in PFS or OS and that oral may be favorable option for cancer patients in a palliative setting.

Previously, studies have shown conflicting results leading to a disadvantage for oral chemotherapy being used clinically. However, our data imply that oral administration of etoposide may be a good alternative for GEP-NEN G3 patients. Prospective studies are needed in order to validate and establish a consensus on the efficacy of oral chemotherapy and whether this option is being overlooked with regard to cost effectiveness, safety and patient preference.
